# High prevalence of non-steroidal anti-inflammatory drug use among acute kidney injury survivors in the southern community cohort study

**DOI:** 10.1186/s12882-016-0411-7

**Published:** 2016-11-24

**Authors:** Loren Lipworth, Khaled Abdel-Kader, Jennifer Morse, Thomas G. Stewart, Edmond K. Kabagambe, Sharidan K. Parr, Kelly A. Birdwell, Michael E. Matheny, Adriana M. Hung, William J. Blot, T. Alp Ikizler, Edward D. Siew

**Affiliations:** 1 Department of Medicine, Division of Epidemiology, Vanderbilt University Medical Center, Nashville, TN USA; 2 Vanderbilt Center for Kidney Disease (VCKD) and Integrated Program for Acute Kidney Injury Research (VIP-AKI), Nashville, TN USA; 3 Department of Medicine, Division of Nephrology and Hypertension, Vanderbilt University Medical Center, Nashville, TN USA; 4 Department of Biostatistics, Vanderbilt University Medical Center, Nashville, TN USA; 5 Tennessee Valley Healthcare System (TVHS) VA Medical Center, TVHS Geriatric Research Education and Clinical Centers (GRECC), Veteran’s Health Administration, Nashville, TN USA

**Keywords:** Acute kidney injury, NSAIDs

## Abstract

**Background:**

Non-steroidal anti-inflammatory drugs (NSAIDs) are widely used and have been linked to acute kidney injury (AKI), chronic kidney disease (CKD) and cardiovascular disease (CVD). Patients who survive an AKI episode are at risk for future adverse kidney and cardiovascular outcomes. The objective of our study was to examine the prevalence and predictors of NSAID use among AKI survivors.

**Methods:**

The Southern Community Cohort Study is a prospective study of low-income adults aged 40–79 in the southeastern US. Through linkage with Centers for Medicare and Medicaid Services, 826 participants with an AKI diagnosis (ICD-9 584.5-584.9) at any age prior to cohort enrollment were identified. At baseline, data were collected on regular use of prescription and over-the-counter NSAIDs, as well as demographic, medical and other characteristics. Additional comorbidities were ascertained via linkage with CMS or the US Renal Data System.

**Results:**

One hundred fifty-four AKI survivors (19%) reported regular NSAID use at cohort enrollment (52 prescription, 81 OTC, 21 both) and the percentage of NSAID users did not vary by time since AKI event. Over 58% of users were taking NSAIDS regularly both before and after their AKI event. Hypertension (83%), arthritis (71%), heart failure (44%), CKD (36%) and diabetes (35%) were prevalent among NSAID users. In a multivariable model, history of arthritis (OR: 3.00; 95% CI: 1.92, 4.68) and acetaminophen use (OR: 2.43; 95% CI: 1.50, 3.93) were significantly associated with NSAID use, while prevalent CKD (OR: 0.63; 95% CI: 0.41, 0.98) and diabetes (OR: 0.44; 95% CI: 0.29, 0.69) were significantly inversely associated.

**Conclusions:**

NSAID use among AKI survivors is common and highlights the need to understand physician and patient decision-making around NSAIDs and to develop effective strategies to reduce NSAID use in this vulnerable population.

**Electronic supplementary material:**

The online version of this article (doi:10.1186/s12882-016-0411-7) contains supplementary material, which is available to authorized users.

## Background

Acute kidney injury (AKI) is one of the fastest growing conditions affecting the kidney and a risk factor for accelerated loss of kidney function and cardiovascular disease (CVD) [1–3]. Improving outcomes among the growing population of AKI survivors requires identifying modifiable risk factors to reduce these events. Non-steroidal anti-inflammatory drugs (NSAIDs) are the most commonly used analgesics worldwide and an established risk factor for AKI [4, 5], chronic kidney disease (CKD) [6], and CVD [7]. The American Society of Nephrology (ASN) Quality and Patient Safety (QPS) Task Force highlighted the avoidance of NSAIDs as a high priority area “most open” to improvement in patients with kidney disease [8]. Similarly, the US Food and Drug Administration (FDA) recently strengthened its warning regarding the link between NSAIDs and major vascular events, particularly among those with cardiovascular risk factors [9].

Despite growing attention to the dangers of NSAID use among high-risk populations, use is common among patients with CKD, ranging between 4–31% depending on the populations studied [10, 11]. However, to our knowledge, no studies to date have examined their use in patients who have experienced AKI. Given the high prevalence of CKD in this population and evidence linking AKI to future kidney and cardiovascular outcomes [12, 13], examining NSAID use among survivors of AKI is important for identifying the magnitude of the gap between patient and provider practice and guideline recommendations. To examine the frequency of NSAID use among AKI survivors in a low socioeconomic population which carries a disproportionate risk of CKD and end-stage renal disease [14], as well as factors associated with NSAID use among AKI survivors, we leveraged detailed analgesic intake information collected from participants in the Southern Community Cohort Study (SCCS), a large prospective cohort of low-income adults in the southeastern US.

## Methods

The SCCS is an ongoing, prospective cohort study designed to study incident cancer and other chronic diseases. From 2002 through 2009, the SCCS enrolled nearly 86,000 adults aged 40–79 years, two-thirds black, residing in 12 states in the southeastern US. The study design and methods have been described in detail [15]. Approximately 86% of participants were enrolled through community health centers (CHC), institutions providing primary health and preventive care services in medically underserved areas and thus serving generally low-income populations [14]. The remaining 14% were recruited via mail-based general population sampling. For all participants, data on socioeconomic, demographic and lifestyle characteristics, as well as personal medical and medication use history, were ascertained at cohort enrollment using the same questionnaire, administered via standardized computer-assisted personal interview for CHC participants, and via self-administered mailed questionnaire for general population participants (questionnaire at www.southerncommunitystudy.org). SCCS participants provided written informed consent, and the Institutional Review Boards of Vanderbilt University Medical Center and Meharry Medical College approved all study protocols, which adhere to the Declaration of Helsinki. We adhered to the guidelines and methodology set forth in the Strengthening the Reporting of Observational studies in Epidemiology Statement [16].

### Study population and setting

We restricted our analyses to SCCS participants who had an inpatient or outpatient AKI diagnosis prior to study enrollment, ascertained via linkage of the cohort, using Social Security number, date of birth and first and last name, with Centers for Medicare and Medicaid Services (CMS) Research Identifiable Files from January 1, 1999 through December 31, 2010. Prevalent AKI was defined using inpatient and outpatient medical claims with ICD-9 code 584.5-584.9 within either the Medicare institutional (Medicare Provider Analysis and Review, MEDPAR), Part B carrier or outpatient-based claims files (for those 65 years and older), or the Medicaid Analytic Extract (MAX) Inpatient and Other Services claims files (for those under age 65 years) [17]. For those who had multiple AKI events recorded prior to enrollment, the one closest to enrollment was selected as the index AKI event. A total of 1018 SCCS participants with a diagnosis of AKI prior to enrollment in the cohort (AKI survivors) were identified.

We excluded patients with a diagnosis of end-stage renal disease (ESRD), ascertained by linkage with the United States Renal Data System (USRDS) from January 1, 2002 through September 1, 2012. The USRDS registers ESRD cases certified by a physician diagnosis and filed using a medical evidence report form (to the Medicare ESRD program) or when there is other evidence of chronic dialysis or a kidney transplant irrespective of the glomerular filtration rate [18], providing virtually complete ascertainment of all persons in the US receiving treatment for ESRD. After exclusion of 192 individuals with a diagnosis of ESRD prior to SCCS enrollment, a total of 826 AKI survivors were included in the current analyses (see Additional file 1: Figure S1).

### Ascertainment of regular analgesic use

At cohort enrollment, participants were asked: “In the past year, have you taken any of the following medications regularly? By regularly, we mean at least two times per week for one month or more.” Listed medications included the names of both prescription (Celebrex, Vioxx, Bextra) and over-the-counter (OTC) NSAIDs (including Advil, Motrin, Aleve, Ibuprofen, etc.) and other analgesic medications, such as low-dose aspirin, regular aspirin (Anacin, Bayer, Bufferin, Excedrin, etc.) and acetaminophen (Tylenol). Those who responded affirmatively were asked additional follow-up questions regarding number of years of regular use and average number of pills per week for each medication. From data on self-reported duration of regular NSAID use and date of index AKI CMS claim, a variable was created to indicate whether the participant started taking NSAIDs before or after the index AKI event.

For the purposes of estimating the prevalence of NSAID use, a study participant was classified as a regular NSAID user if he or she reported regularly using either prescription or OTC NSAIDs. If the participant reported non-use or unknown use, he or she was classified as NSAID non-user. This classification scheme provides what we consider a lower bound on the prevalence of NSAID use in the study population. For the purposes of estimating relative measures of NSAID use, observations missing the outcome were incorporated into the analysis via multiple imputation, a method for missing data.

### Assessment of comorbidities

Comorbidities were assessed in two ways. At baseline, participants were asked whether they had ever received a physician diagnosis of hypertension, diabetes, arthritis, myocardial infarction/coronary artery bypass graft (MI/CABG), and high cholesterol. We ascertained a history of CKD and heart failure by linkage of the cohort with CMS and were defined as the occurrence, prior to the date of SCCS enrollment, of a medical claim with ICD-9 code 585.1-585.5 or 428.x, respectively.

### Statistical analysis

Participants’ baseline characteristics were summarized as medians (and corresponding 25th and 75th percentiles) for continuous variables and counts (%) for categorical variables. The primary estimates of prevalence used the “lower bound” definition of NSAID use. Relative measures of use were estimated via a multivariable model with the “yes/no/missing” definition of NSAID use as the outcome. The multivariable analysis of NSAID use began by specifying a priori a set of covariates believed to be predictors of the outcome. The set of covariates included demographic variables (age, sex, race, education, income, recruitment source), indicators of comorbidities (heart failure, hypertension, diabetes, arthritis, CKD), acetaminophen use and a variable for time since prior AKI. The multivariable associations of NSAID use and the list of covariates were estimated with a logistic regression model. All available data were incorporated into the model by using multiple imputation with predictive mean matching to account for uncertain outcome data (234 patients) or predictor data (52 patients); 300 imputed datasets were generated. After imputation, only two outcome categories remained (yes/no) for the logistic regression model. We also conducted a sensitivity analysis using the complete-case definition (unknown NSAID users excluded), the lower bound definition (unknown NSAID use considered as non-use, as described above) and multiple imputation using only participants with an observed outcome. All analyses were performed in R, free software for statistical computing, version 3.2.5 (http://www.r-project.org/).

## Results

Among 826 SCCS participants with a history of AKI prior to enrollment, the median time between AKI event and SCCS enrollment was 13.4 months (range 0.03–121.02), and approximately 67% experienced their AKI event within the prior two years. Overall, 19% (*n* = 154) of AKI survivors reported regular use of NSAIDs at cohort enrollment, and the percentage of NSAID users did not vary substantially by time since AKI event (Table 1). Of the 154 NSAID users, 52 (34%) reported use of prescription NSAIDs only, 81 (53%) reported OTC NSAID use only, and the remaining 21 (14%) reported using both prescription and OTC NSAIDs.

**Table 1:** Non-steroidal anti-inflammatory drug (NSAID) use among 826 acute kidney injury (AKI) survivors in the Southern Community Cohort Study, by time since most recent AKI event prior to cohort enrollment

Timing of prior AKI (years)	Patients, *N* (%)	Taking NSAID, *N* (%)^a^
0.25	114 (13.8)	19 (16.7)
0.5	220 (26.6)	39 (17.7)
1	369 (44.7)	71 (19.2)
1.5	488 (59.1)	97 (19.9)
2	554 (67.1)	107 (19.3)
3	697 (84.4)	131 (18.8)
4	754 (91.3)	141 (18.7)
5	783 (94.8)	148 (18.9)
All records	826 (100)	154 (18.6)

^a^Either prescription or OTC NSAID or both

For prescription NSAIDs, the median duration of regular use was 2.0 years (25th percentile 0.5, 75th percentile 3.0) and the median number of pills per week was 7.0 (7.0, 14.0); corresponding numbers for OTC NSAIDs were 3.0 (1.0, 10.0) years and 7.5 (4.0, 14.8) pills per week, respectively. Among NSAID users, 33.1% (51/154) reported regular NSAID use starting after their AKI event, whereas 58.4% (90/154) reported regular NSAID use starting prior to their AKI claim and continuing to enrollment (data not shown). Thirteen participants (8.4%) reported regular NSAID use, but did not report when the use started.

Characteristics of AKI survivors who used NSAIDs (*N* = 154) and those who did not use NSAIDs (*N* = 438) or had unknown NSAID use (*N* = 234) are presented in Table 2. For both users and non-users of NSAIDs, median age at SCCS enrollment was approximately 58 years, slightly more than half were women, and approximately 75% had an annual household income < $15,000. Over 90% reported some form of health insurance coverage and 50% reported having seen a doctor within the past month. Among NSAID users, 53% were black compared to 64% of non-NSAID users, and NSAID users were more likely than non-NSAID users to have an education level greater than high-school (38% versus 27%). There were no material differences in any of the examined characteristics between those who reported non-use of NSAIDs and those with unknown NSAID use.

**Table 2:** Characteristics of users and non-users of non-steroidal anti-inflammatory drugs (NSAIDs) among 826 acute kidney injury (AKI) survivors in the Southern Community Cohort Study

Characteristic	NSAID users^a,c^ *N* = 154	NSAID non-users^a,c^ *N* = 438	Unknown NSAID^a,c^ *N* = 234	Total^a,c^ *N* = 826	Median difference between NSAID users and non-users^d^
Age at enrollment, years	57.5 (50.0, 66.0)	58.0 (50.0, 66.0)	57.0 (50.0, 65.0)	57.0 (50.0, 66.0)	−0.5 (−4.1, 1.6)
Recruitment source					
CHC	111 (72)	406 (93)	218 (93)	735 (89)	
General population	43 (28)	32 (7)	16 (7)	91 (11)	
Race					
Black	79 (53)	280 (64)	176 (77)	535 (66)	
White	66 (44)	137 (31)	42 (18)	245 (30)	
Other	4 (3)	20 (5)	12 (5)	36 (4)	
Male	66 (43)	215 (49)	108 (46)	389 (47)	−6.2 (−15.8, 3.3)
Income < $15,000	113 (75)	336 (76)	190 (80)	629 (77)	1.1 (−7.3, 9.5)
Education					
< High school	46 (31)	177 (41)	105 (47)	328 (41)	
High school	46 (31)	141 (32)	70 (32)	257 (32)	
> High school	57 (38)	118 (27)	47 (21)	222 (28)	
Insurance coverage	143 (93)	398 (91)	204 (93)	204 (93)	2.4 (−2.8, 7.6)
Most recent doctor visit, months					
0	53 (36)	185 (43)	86 (40)	324 (41)	
1	56 (38)	133 (31)	79 (37)	268 (34)	
2	20 (14)	33 (8)	24 (11)	77 (10)	
3	19 (13)	76 (18)	26 (12)	121 (15)	
BMI, kg/m^2^	30.0 (26.1, 36.7)	29.4 (25.6, 36.0)	29.2 (24.2, 35.9)	29.4 (25.3, 36.1)	0.7 (−0.5, 1.9)
Regular use of:					
Low-dose aspirin	66 (44)	162 (37)	59 (27)	287 (36)	
Regular aspirin	34 (24)	64 (15)	41 (19)	139 (18)	
Acetaminophen	50 (35)	62 (14)	18 (9)	130 (17)	
NSAID source^b^					
Prescription	52 (34)	-	-	52 (34)	
OTC	81 (53)	-	-	81 (53)	
Both	21 (14)	-	-	21 (14)	
Diabetes	54 (35)	233 (53)	114 (51)	401 (49)	−18.1 (−27.4, −8.8)
Hypertension	128 (83)	348 (79)	181 (81)	657 (81)	3.7 (−3.8, 11.1)
Myocardial infarction	29 (19)	117 (27)	52 (23)	198 (24)	−7.8 (−15.7, 0.2)
Arthritis	109 (71)	187 (43)	108 (48)	404 (50)	28.1 (19.1, 37.1)
Ulcer	41 (27)	77 (18)	38 (17)	156 (19)	9.0 (0.7, 17.3)
Lupus	4 (3)	6 (1)	4 (2)	14 (2)	1.2 (−2.0, 4.4)
High cholesterol	78 (51)	223 (51)	104 (47)	405 (50)	−0.1 (−9.3, 9.2)
Heart failure	68 (44)	224 (51)	119 (51)	411 (50)	−7.0 (−16.6, 2.6)
Chronic kidney disease	55 (36)	215 (49)	91 (39)	361 (44)	−13.4 (−22.7, −4.0)

^a^Data presented as median (25th, 75th percentile) for continuous variables and *N* (%) for categorical variables

^b^Percentages sum to >100% due to rounding

^c^n may not sum to 826 due to missing data

^d^Presented as median difference for continuous variables and proportion difference for categorical variables

The prevalence of hypertension (~80%) and hypercholesterolemia (~50%) were similarly high among NSAID users and non-users. Approximately one-third of NSAID users reported a history of diabetes, one in five reported a prior history of MI and 36% and 44% had a previous CMS claim for CKD and heart failure, respectively. A higher proportion of NSAID users reported a diagnosis of arthritis compared to nonusers (71% versus 43%) and NSAID users were more likely to also report regular use of low-dose aspirin (44% vs. 37%), regular-dose aspirin (24% vs. 15%) and acetaminophen (35% vs. 14%).

Table 3 presents multivariable logistic regression-derived odds ratios (OR) and 95% confidence intervals (CI) for the association between various participant characteristics and NSAID use among AKI survivors. Neither sex [OR (male): 0.84; 95% CI: 0.54, 1.30], race [OR (black): 0.81; 95% CI: 0.53, 1.24] nor education [OR (≥high school): 1.51; 95% CI: 0.96, 2.40] was statistically significantly associated with odds of NSAID use. Patients with a history of arthritis had significantly increased odds of using NSAIDs (OR: 3.00; 95% CI: 1.92, 4.68), as did users of acetaminophen (OR: 2.43; 95% CI: 1.50, 3.93), while diabetes (OR: 0.44; 95% CI: 0.29, 0.69) and CKD (OR: 0.63; 95% CI: 0.41-0.98) were inversely associated with NSAID use. Time since index AKI episode was marginally statistically significant, while hypertension, heart failure and participant age were not significant predictors of NSAID use.

**Table 3:** Multivariable logistic regression-derived odds ratios (OR) and 95% confidence intervals (CI) for the association between participant characteristics and use of non-steroidal anti-inflammatory drugs ﻿(NSAIDs) among 826 acute kidney injury (AKI) survivors in the Southern Community Cohort Study (SCCS)

Associated factors	OR^a^	95% CI
Enrollment Age^b^	0.99	(0.97, 1.02)
Male sex	0.84	(0.54, 1.30)
Black race	0.81	(0.53, 1.24)
Income, >$15,000 vs. <$15,000	0.88	(0.52, 1.48)
Education, > high school vs. < high school	1.51	(0.96, 2.40)
Arthritis	3.00	(1.92, 4.68)
Hypertension	1.43	(0.82, 2.49)
Diabetes	0.44	(0.29, 0.69)
Years since AKI^b^	0.92	(0.80, 1.05)
Chronic kidney disease	0.63	(0.41, 0.98)
Heart failure	0.97	(0.63, 1.50)
Acetaminophen	2.43	(1.50, 3.93)

^a^All variables were entered in the model simultaneously, along with a variable indicating SCCS recruitment source (CHC vs. General Population)

^b^OR per 1 year increase

Sensitivity analyses excluding from the models those with unknown NSAID use or implementing alternate multiple imputation methods yielded similar estimates of association (see Additional file 2: Figure S2).

## Discussion

Survivors of AKI are at increased risk for future kidney dysfunction [19–21], cardiovascular complications [3, 21], and death [22, 23]. In this study, we demonstrate that nearly one in five AKI survivors were using NSAIDs regularly and that the prevalence remained consistently high regardless of time since the most recent AKI episode. Alarmingly, over one-half of NSAID users were taking these medications before and after the index AKI episode. Further, we demonstrate that in this predominantly low-income population, use of NSAIDs following AKI was somewhat more likely among participants with arthritis and those without diabetes.

The path to ESRD is often non-linear and marked by one or more AKI episodes [24, 25]. The incidence of AKI is also rising by approximately 10% per year [2], highlighting a growing population of survivors in need of follow-up care. While optimal care remains to be defined, little debate exists regarding the adverse effects of chronic NSAID use on the kidney and cardiovascular systems. The use of NSAIDs in patients at risk or with established CKD is a well-recognized problem, and was recently emphasized by the ASN QPS Task Force as one of five highest impact evidence-based areas in need of improvement [26]. Patients who experience AKI, particularly when superimposed on CKD (36% of NSAID users in our cohort), are at highest risk for ESRD and thus represent a subgroup in whom preventing future loss of kidney function may be especially important [27, 28]. One potential mechanism for this increased risk may be through recurrent AKI. We have recently demonstrated that 25% of AKI survivors are re-hospitalized with another AKI event within a year of discharge [28]. This study adds to the literature by pointing out a potentially modifiable risk factor for preventing recurrent AKI.

Even in the absence of CKD, AKI has been demonstrated to lead to impaired sodium handling and autoregulatory capacity [29, 30]. When coupled with NSAID use, which can increase sodium retention and blood pressure [31, 32], these derangements may increase susceptibility to recurrent AKI [28], hypertension [1], and cardiovascular events [3] in AKI survivors. The FDA has also recently strengthened its warning regarding NSAIDs and major vascular events, particularly among those with traditional cardiovascular risk factors [9]. Diabetes, MI, hypertension and heart failure were all common among AKI survivors in our study. Participants with diabetes were less likely to use NSAIDs, perhaps due to awareness of CKD risk among diabetic patients. Alternatively, NSAIDs are not considered the optimal treatment for neuropathic pain, which is common among diabetics. Nevertheless, the combination of these comorbidities, NSAID use and AKI may further enhance the vulnerability of this population to subsequent adverse kidney and cardiovascular events. More proximal use of NSAIDs may also impact recovery from AKI. This is of particular concern since approximately 17% of patients experiencing AKI within the past 3 or 6 months were regularly taking NSAIDs.

A strong predictor of NSAID use in our study was a history of arthritis. Although arthritis was self-reported and likely included a mix of rheumatic disease, including rheumatoid arthritis, osteoarthritis and ankylosing spondylitis, osteoarthritis was the likely indication for NSAID use for many of these patients. Osteoarthritis prevalence in the US is rising [33], and while NSAIDs are effective anti-inflammatory and analgesic agents, re-examination of their risks and benefits has prompted decreased use in this patient population [34]. Current clinical guidelines for osteoarthritis management recommend acetaminophen for pain relief as first-line therapy ahead of oral NSAIDs [35], with rising pressure in some countries to make all NSAIDs available by prescription only [36]. Increasing awareness of NSAID risks among AKI survivors with arthritis, as well as other serious comorbidities, may be especially important in light of concurrent efforts to limit opioid use among these patients.

The prevalence of NSAID use among AKI survivors was comparable to that in the SCCS cohort overall (23%), and in a subset of the cohort restricted to those with at least one CMS claim prior to SCCS enrollment (23%; data not shown). Although recruited primarily through CHCs and thus with similar healthcare access regardless of race and income, the high rates of post-AKI NSAID use in this low socioeconomic status population are concerning in light of well-described disparities in the development and progression of CKD among the poor [37, 38]. Without adjusting for other covariates, black AKI survivors in our study were less likely to use NSAIDs compared to whites; however, the difference is not statistically significant when adjusting for socioeconomic characteristics, education and comorbidities. This also mirrors the pattern of NSAID use observed in the SCCS population overall, in which 20% of blacks reported NSAID use compared to 28% of whites. The prevalence of CKD at baseline was somewhat higher among blacks compared to whites (46% vs. 41%), and it is possible that physicians may have greater recognition of CKD risk among blacks and thus be less likely to prescribe NSAIDs to them. However, OTC NSAID use was also less common among blacks, and we cannot rule out under-treatment of pain in this group.

There is a clear need to develop strategies to reduce unnecessary NSAID use among AKI survivors, especially as rates of regular NSAID use in this study were two to three times greater than reported in studies of patients with CKD [11, 39]. The higher prevalence of use may be explained in part by our capture of both prescription and OTC exposure, as well as by the geographic catchment area of the SCCS, which overlaps with regions shown to have higher utilization of prescriptions with potentially harmful drug-disease interactions [40], including in CKD. These findings support a critical role for improving education and awareness of the potential hazard of NSAID use. Research has already demonstrated that patient awareness of CKD is low [41], and awareness among AKI survivors of the potential nephrotoxicity of NSAIDs may be similarly low [42]. As most survivors of AKI will not be seen by a nephrology provider [43], the burden of risk reduction falls to patients and their primary providers. The similar prevalence of NSAID use among AKI survivors compared to the general SCCS cohort suggests that increasing awareness among both will be equally important as about 50% of NSAID use in our study was by prescription.

Strengths of our study include capture of both prescription and non-prescription NSAID use and its examination within a lower socioeconomic patient population with a high burden of risk factors for both CVD and kidney disease. While several million prescriptions for NSAIDs are estimated to be written annually in the US [44], non-prescription, OTC use is likely more common requiring capture of information of both sources to understand the full burden of use [45]. Moreover, comparison of black and white participants of similar socioeconomic status and comparable health care access minimizes confounding effects related to socioeconomic status.

Limitations of the study include the use of administrative data to identify prevalent AKI. However, multiple studies have demonstrated that administrative codes are specific and likely to capture more severe AKI [46], highlighting the concerning prevalence of NSAID use among the subset of AKI survivors at highest risk for experiencing poor renal and cardiovascular outcomes [47]. Because our cohort is likely enriched for more severe AKI, our findings also likely represent an underestimate of the overall burden of NSAID use among all survivors of AKI. Similarly, the known sensitivity limitations of administrative codes for detecting AKI precluded our ability to perform a meaningful analysis of the association of NSAID use with future adverse events. However, the prevalence of use in this population does set the stage for further studies to determine the level of concern. The study also relied on self-report of NSAID use, and the wording of the questionnaire defined regular use as use at least two times per week for one month or more, which could result in some misclassification, particularly with respect to specific type of analgesic used or duration of regular use. Although we accounted for several participant characteristics or comorbidities (e.g. arthritis) that may be associated with NSAID use, there remains a possibility of residual confounding by indication from other comorbidities, CKD stage or underlying causes or intensity of pain that may be associated with NSAID use and for which information was not available. Lastly, our study is generalizable only to patients who survived an episode of AKI without ESRD. Moreover, participants were drawn from a population in the southeastern US with generally low socioeconomic status, thus the findings of this study may not be generalizable to other populations.

## Conclusion

In summary, regular NSAID use is common (20%) among socioeconomically disadvantaged AKI survivors. The high prevalence of an avoidable risk factor in a group at high risk for both kidney and cardiovascular events is concerning and underscores the need to better understand the association between NSAID use in this population and future adverse events and in whom the risks are highest, as well as the reasons for use and effective strategies to improve education and awareness among physicians, patients and caregivers.

## Additional files

Additional file 1: Figure S1. Study flow chart (PPT 111 kb)
Additional file 2: Figure S2. Comparison of multivariable logistic-regression derived odds ratios and 95% confidence intervals from all sensitivity analyses, using the complete-case definition (unknown NSAID users excluded), the lower bound definition (unknown NSAID use considered as non-use), and multiple imputation using only participants with an observed outcome (PNG 82 kb)

## Abbreviations

AKI: Acute kidney injury
ASN: American Society of Nephrology
CHC: Community health centers
CI: Confidence interval
CKD: Chronic kidney disease
CMS: Centers for Medicare and Medicaid Services
CVD: Cardiovascular disease
ESRD: End-stage renal disease
FDA: Food and Drug Administration
MI/CABG: Myocardial infarction/coronary artery bypass graft
NSAID: Non-steroidal anti-inflammatory drug
OR: Odds ratio
OTC: Over-the-counter
QPS: Quality and Patient Safety
SCCS: Southern Community Cohort Study
USRDS: United States Renal Data System

## References

[1] Hsu CY, Hsu RK, Yang J, Ordonez JD, Zheng S, Go AS (2016). Elevated BP after AKI. J Am Soc Nephrol.

[2] Hsu RK, McCulloch CE, Dudley RA, Lo LJ, Hsu CY (2013). Temporal changes in incidence of dialysis-requiring AKI. J Am Soc Nephrol.

[3] Wu VC, Wu CH, Huang TM, Wang CY, Lai CF, Shiao CC, Chang CH, Lin SL, Chen YY, Chen YM (2014). Long-term risk of coronary events after AKI. J Am Soc Nephrol.

[4] Leonard CE, Freeman CP, Newcomb CW, Reese PP, Herlim M, Bilker WB, Hennessy S, Strom BL (2012). Proton pump inhibitors and traditional nonsteroidal anti-inflammatory drugs and the risk of acute interstitial nephritis and acute kidney injury. Pharmacoepidemiol Drug Saf.

[5] Schneider AG, Bellomo R, Bagshaw SM, Glassford NJ, Lo S, Jun M, Cass A, Gallagher M (2013). Choice of renal replacement therapy modality and dialysis dependence after acute kidney injury: a systematic review and meta-analysis. Intensive Care Med.

[6] Gooch K, Culleton BF, Manns BJ, Zhang J, Alfonso H, Tonelli M, Frank C, Klarenbach S, Hemmelgarn BR (2007). NSAID use and progression of chronic kidney disease. Am J Med.

[7] Bhala N, Emberson J, Merhi A, Abramson S, Arber N, Baron JA, Bombardier C, Cannon C, Farkouh ME, FitzGerald GA (2013). Vascular and upper gastrointestinal effects of non-steroidal anti-inflammatory drugs: meta-analyses of individual participant data from randomised trials. Lancet.

[8] Williams AW, Dwyer AC, Eddy AA, Fink JC, Jaber BL, Linas SL, Michael B, O'Hare AM, Schaefer HM, Shaffer RN (2012). Critical and honest conversations: the evidence behind the “Choosing Wisely” campaign recommendations by the American Society of Nephrology. Clin J Am Soc Nephrol.

[9] Food and Drug Administration strengthens warnings that non-aspirin nonsteroidal anti-inflammatory drugs (NSAIDs) can cause heart attacks or strokes. [http://www.fda.gov/downloads/Drugs/DrugSafety/UCM453941.pdf]. Accessed 21 Nov 2016.

[10] Adams RJ, Appleton SL, Gill TK, Taylor AW, Wilson DH, Hill CL (2011). Cause for concern in the use of non-steroidal anti-inflammatory medications in the community--a population-based study. BMC Fam Pract.

[11] Plantinga L, Grubbs V, Sarkar U, Hsu CY, Hedgeman E, Robinson B, Saran R, Geiss L, Burrows NR, Eberhardt M (2011). Nonsteroidal anti-inflammatory drug use among persons with chronic kidney disease in the United States. Ann Fam Med.

[12] Coca SG, Singanamala S, Parikh CR (2012). Chronic kidney disease after acute kidney injury: a systematic review and meta-analysis. Kidney Int.

[13] Hsu CY, Ordonez JD, Chertow GM, Fan D, McCulloch CE, Go AS (2008). The risk of acute renal failure in patients with chronic kidney disease. Kidney Int.

[14] Lipworth L, Mumma MT, Cavanaugh KL, Edwards TL, Ikizler TA, Tarone RE, McLaughlin JK, Blot WJ (2012). Incidence and predictors of end stage renal disease among low-income blacks and whites. PLoS One.

[15] Signorello LB, Hargreaves MK, Blot WJ (2010). The Southern Community Cohort Study: investigating health disparities. J Health Care Poor Underserved.

[16] von Elm E, Altman DG, Egger M, Pocock SJ, Gotzsche PC, Vandenbroucke JP (2008). The Strengthening the Reporting of Observational Studies in Epidemiology (STROBE) statement: guidelines for reporting observational studies. J Clin Epidemiol.

[17] Waikar SS, Wald R, Chertow GM, Curhan GC, Winkelmayer WC, Liangos O, Sosa MA, Jaber BL (2006). Validity of International Classification of Diseases, Ninth Revision, Clinical Modification Codes for Acute Renal Failure. J Am Soc Nephrol.

[18] United States Renal Data System (2014). USRDS 2014 Annual Data Report: Atlas of Chronic Kidney Disease and End-Stage Renal Disease in the United States.

[19] James MT, Ghali WA, Knudtson ML, Ravani P, Tonelli M, Faris P, Pannu N, Manns BJ, Klarenbach SW, Hemmelgarn BR (2011). Associations between acute kidney injury and cardiovascular and renal outcomes after coronary angiography. Circulation.

[20] James MT, Ghali WA, Tonelli M, Faris P, Knudtson ML, Pannu N, Klarenbach SW, Manns BJ, Hemmelgarn BR (2010). Acute kidney injury following coronary angiography is associated with a long-term decline in kidney function. Kidney Int.

[21] Wald R, Quinn RR, Luo J, Li P, Scales DC, Mamdani MM, Ray JG (2009). Chronic dialysis and death among survivors of acute kidney injury requiring dialysis. JAMA.

[22] James MT, Hemmelgarn BR, Wiebe N, Pannu N, Manns BJ, Klarenbach SW, Tonelli M (2010). Glomerular filtration rate, proteinuria, and the incidence and consequences of acute kidney injury: a cohort study. Lancet.

[23] Newsome BB, Warnock DG, McClellan WM, Herzog CA, Kiefe CI, Eggers PW, Allison JJ (2008). Long-term risk of mortality and end-stage renal disease among the elderly after small increases in serum creatinine level during hospitalization for acute myocardial infarction. Arch Intern Med.

[24] Li L, Astor BC, Lewis J, Hu B, Appel LJ, Lipkowitz MS, Toto RD, Wang X, Wright JT, Greene TH (2012). Longitudinal progression trajectory of GFR among patients with CKD. Am J Kidney Dis.

[25] O'Hare AM, Batten A, Burrows NR, Pavkov ME, Taylor L, Gupta I, Todd-Stenberg J, Maynard C, Rodriguez RA, Murtagh FE (2012). Trajectories of kidney function decline in the 2 years before initiation of long-term dialysis. Am J Kidney Dis.

[26] Ishani A, Xue JL, Himmelfarb J, Eggers PW, Kimmel PL, Molitoris BA, Collins AJ (2009). Acute kidney injury increases risk of ESRD among elderly. J Am Soc Nephrol.

[27] Hsu CY, Chertow GM, McCulloch CE, Fan D, Ordonez JD, Go AS (2009). Nonrecovery of kidney function and death after acute on chronic renal failure. Clin J Am Soc Nephrol.

[28] Siew ED, Parr SK, Abdel-Kader K, Eden SK, Peterson JF, Bansal N, Hung AM, Fly J, Speroff T, Ikizler TA (2016). Predictors of Recurrent AKI. J Am Soc Nephrol.

[29] Conger JD, Robinette JB, Schrier RW (1988). Smooth muscle calcium and endothelium-derived relaxing factor in the abnormal vascular responses of acute renal failure. J Clin Invest.

[30] Pechman KR, De Miguel C, Lund H, Leonard EC, Basile DP, Mattson DL (2009). Recovery from renal ischemia-reperfusion injury is associated with altered renal hemodynamics, blunted pressure natriuresis, and sodium-sensitive hypertension. Am J Physiol Regul Integr Comp Physiol.

[31] Brater DC, Harris C, Redfern JS, Gertz BJ (2001). Renal effects of COX-2-selective inhibitors. Am J Nephrol.

[32] Gurwitz JH, Avorn J, Bohn RL, Glynn RJ, Monane M, Mogun H (1994). Initiation of antihypertensive treatment during nonsteroidal anti-inflammatory drug therapy. JAMA.

[33] Ong KL, Wu BJ, Cheung BM, Barter PJ, Rye KA (2013). Arthritis: its prevalence, risk factors, and association with cardiovascular diseases in the United States, 1999 to 2008. Ann Epidemiol.

[34] Ausiello JC, Stafford RS (2002). Trends in medication use for osteoarthritis treatment. J Rheumatol.

[35] National Institute for Health and Clinical Excellence (2014). National Clinical Guideline. Osteoarthritis: Care and Management in Adults.

[36] Sibbald B (2006). Ibuprofen redux. CMAJ.

[37] Hossain MP, Goyder EC, Rigby JE, El Nahas M (2009). CKD and poverty: a growing global challenge. Am J Kidney Dis.

[38] Nicholas SB, Kalantar-Zadeh K, Norris KC (2015). Socioeconomic disparities in chronic kidney disease. Adv Chronic Kidney Dis.

[39] Allen AS, Forman JP, Orav EJ, Bates DW, Denker BM, Sequist TD (2011). Primary care management of chronic kidney disease. J Gen Intern Med.

[40] Zhang Y, Baicker K, Newhouse JP (2010). Geographic variation in the quality of prescribing. N Engl J Med.

[41] Cavanaugh KL, Ikizler TA (2013). Acknowledging kidney disease: is ignorance salubrious?. Am J Kidney Dis.

[42] Parr SK, Wild MG, Levea S, Ikizler TA, Siew ED, Cavanaugh KL. ASN Abstract #591, FR_PO498 Assessing Patient Awareness in Moderate to Severe Acute Kidney Injury. 2015.

[43] Siew ED, Peterson JF, Eden SK, Hung AM, Speroff T, Ikizler TA, Matheny ME (2012). Outpatient nephrology referral rates after acute kidney injury. J Am Soc Nephrol.

[44] Laine L (2001). Approaches to nonsteroidal anti-inflammatory drug use in the high-risk patient. Gastroenterology.

[45] Kaufman DW, Kelly JP, Rosenberg L, Anderson TE, Mitchell AA (2002). Recent patterns of medication use in the ambulatory adult population of the United States: the Slone survey. JAMA.

[46] Siew ED, Basu RK, Wunsch H, Shaw AD, Goldstein SL, Ronco C, Kellum JA, Bagshaw SM (2016). Optimizing administrative datasets to examine acute kidney injury in the era of big data: workgroup statement from the 15(th) ADQI Consensus Conference. Can J Kidney Health Dis.

[47] Chawla LS, Amdur RL, Amodeo S, Kimmel PL, Palant CE (2011). The severity of acute kidney injury predicts progression to chronic kidney disease. Kidney Int.

