# Supplementary material for: High prevalence of non-steroidal anti-inflammatory drug use among acute kidney injury survivors in the southern community cohort study
Source: BMC Nephrol. 2016 Nov 24;17:189. doi: 10.1186/s12882-016-0411-7 (PMC5122006; doi:10.1186/s12882-016-0411-7)
Supplement: Additional file 1: Figure S1. — Study flow chart (PPT 111 kb) [file 12882_2016_411_MOESM1_ESM.ppt]

## Slide 1
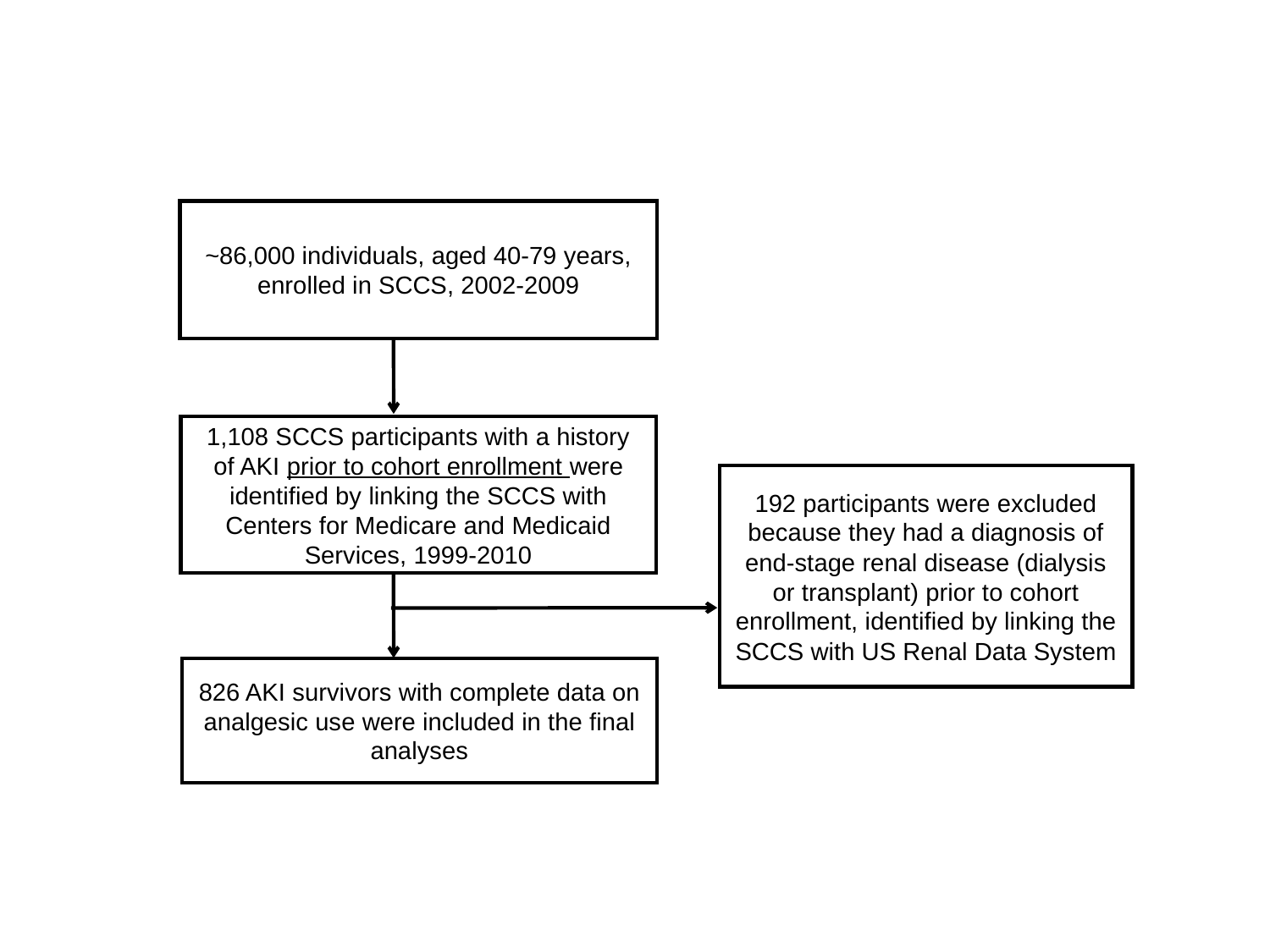

~86,000 individuals, aged 40-79 years, enrolled in SCCS, 2002-2009
1,108 SCCS participants with a history of AKI prior to cohort enrollment were identified by linking the SCCS with Centers for Medicare and Medicaid Services, 1999-2010
192 participants were excluded because they had a diagnosis of end-stage renal disease (dialysis or transplant) prior to cohort enrollment, identified by linking the SCCS with US Renal Data System
826 AKI survivors with complete data on analgesic use were included in the final analyses
