# Supplementary figures and images for: High prevalence of non-steroidal anti-inflammatory drug use among acute kidney injury survivors in the southern community cohort study
Source: BMC Nephrol. 2016 Nov 24;17:189. doi: 10.1186/s12882-016-0411-7 (PMC5122006; doi:10.1186/s12882-016-0411-7)

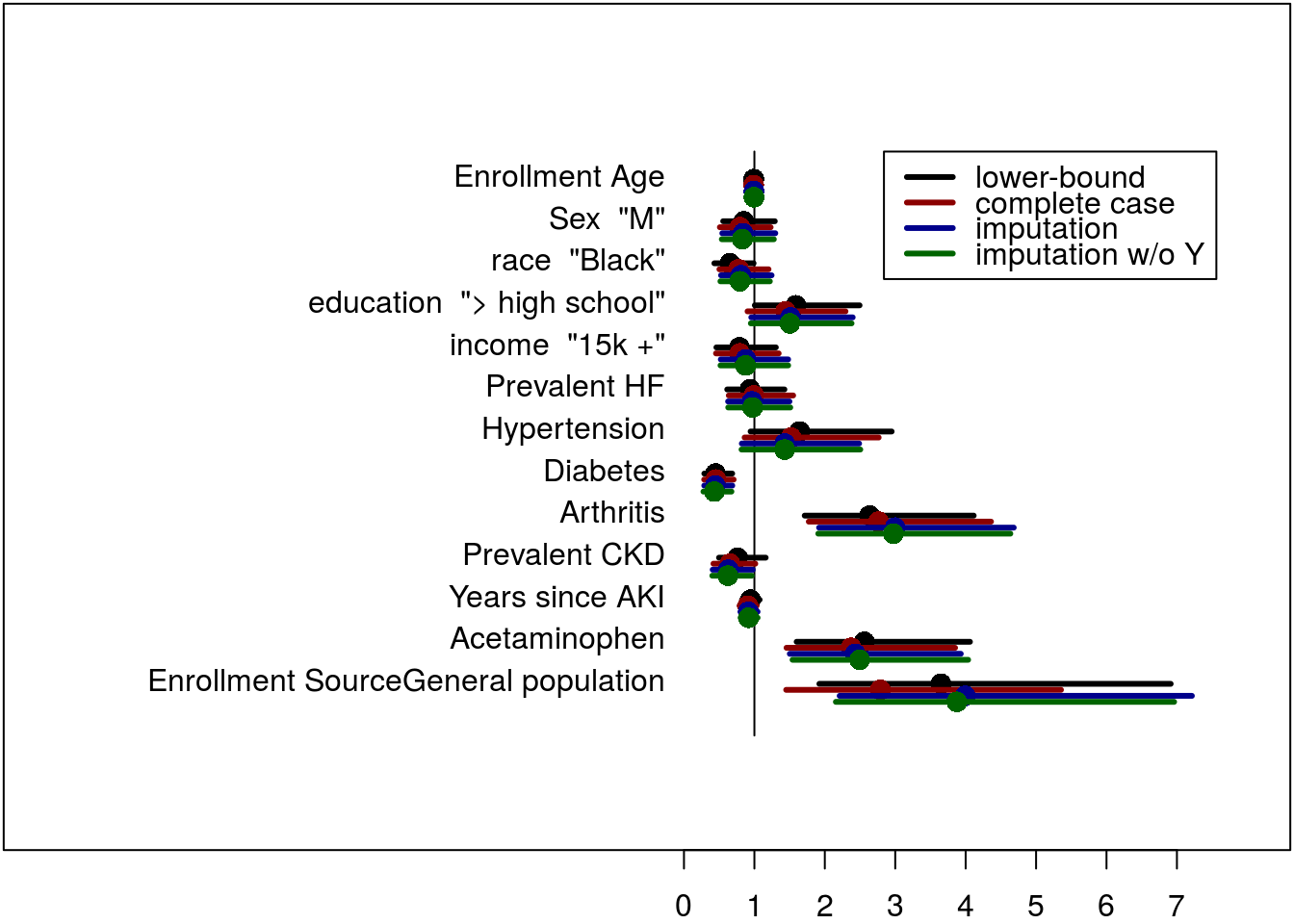

Supplement: Additional file 2: Figure S2. — Comparison of multivariable logistic-regression derived odds ratios and 95% confidence intervals from all sensitivity analyses, using the complete-case definition (unknown NSAID users excluded), the lower bound definition (unknown NSAID use considered as non-use), and multiple imputation using only participants with an observed outcome (PNG 82 kb) [file 12882_2016_411_MOESM2_ESM.png]
